# Menthol Characterizing Flavors in Cigarettes on Sale in England After a Characterizing Flavor Ban: Findings From Sensory and Chemical Assessments

**DOI:** 10.1093/ntr/ntaf064

**Published:** 2025-04-13

**Authors:** Debbie Robson, Christina N Kyriakos, Ann McNeill, Sophie Harrington, Michelle Page, Richard J O’Connor, John Robins, Maciej L Goniewicz

**Affiliations:** Addictions Department, Institute of Psychiatry, Psychology & Neuroscience, King’s College London, London, UK; NIHR ARC South London, UK; Department of Psychiatry, Yale University School of Medicine, New Haven, CT, USA; Department of Primary Care and Public Health, School of Public Health, Imperial College London, London, UK; Addictions Department, Institute of Psychiatry, Psychology & Neuroscience, King’s College London, London, UK; NIHR ARC South London, UK; Addictions Department, Institute of Psychiatry, Psychology & Neuroscience, King’s College London, London, UK; Department of Health Behavior, Roswell Park Comprehensive Cancer Center, Buffalo, NY, USA; Department of Health Behavior, Roswell Park Comprehensive Cancer Center, Buffalo, NY, USA; Addictions Department, Institute of Psychiatry, Psychology & Neuroscience, King’s College London, London, UK; NIHR ARC South London, UK; Department of Health Behavior, Roswell Park Comprehensive Cancer Center, Buffalo, NY, USA

## Abstract

**Introduction:**

In May 2020, the United Kingdom banned menthol as a characteristic flavor in cigarettes. This study aimed to test cigarettes on sale in England in 2021–2022 for menthol and other characterizing flavors, through sensory and chemical testing.

**Aims and Methods:**

Assessments were conducted for 20 cigarette brands (16 tests and four reference products). An untrained consumer panel of 50 people who smoked daily were each randomized to smell one of the two blocks of 10 unburnt cigarettes in duplicate (50 assessments per product). Using the Check-All-That-Apply method, participants assessed the presence of 22 odor attributes, including menthol/mint. For each test and reference cigarette, proportions of assessments that identified menthol/mint, “fruity,” “confectionary” and “non-tobacco” odors were identified and compared accounting for the within-participant duplicate testing. For each cigarette, the content of 34 flavoring chemicals (16 cooling/minty) was analyzed using gas chromatography-mass spectrometry.

**Results:**

Four of the sixteen test cigarette products were more frequently identified by participants as having a menthol/mint odor than reference products *and* had detectable levels of menthol/mint in the chemical tests. For four other test products, there was some discordance between the chemical and sensory assessments. Sensory testing also identified a fruity odor in six test products and a confectionary odor in one test product. The compounds dihydroxyacetone and triacetin were detected above the LLOQ in all products.

**Conclusions:**

Four cigarette products for sale in England in 2021–2022 appeared non-compliant with the ban on menthol as a characterizing flavor in sensory and chemical tests.

**Implications:**

Menthol is known to enhance the appeal of tobacco products, particularly among young people. The subjective nature of determining “characterizing” flavors in tobacco products creates compliance challenges. Our findings suggest that more stringent regulatory policies around flavoring additives used in tobacco products might be necessary. An outright ban on menthol, other minty flavorings, and additives not essential to the manufacturing process could provide clearer guidelines for manufacturers and regulators.

## Introduction

Flavoring agents are added by cigarette manufacturers during the cigarette production process to make the inherent harsh and bitter taste of tobacco smoke more palatable and easier to inhale.^1,2^ Menthol is one of the most commonly used flavoring agents added to tobacco and is used primarily for its chemosensory effects, which create perceptions of a cooling sensation, minty taste, and smell. Menthol activates the transient receptor potential melastatin member 8 (TRPM), which is responsible for its cooling, analgesic, and counter-irritant properties.^3^ Through enhancing the palatability of tobacco smoke and facilitating inhalation, menthol increases the appeal and reinforces smoking behavior, particularly among young people, and enables progression to established use.^1–5^

The World Health Organization Framework Convention on Tobacco Control recommends that Parties ban or restrict ingredients that improve palatability and mask the harshness of tobacco for users, such as menthol.^6^ There are two main legislative approaches that countries have taken: (1) a total ban on flavor additives, and (2) a ban on characterizing flavors. Some countries, for example, Canada, have banned menthol and its analogs as ingredients in tobacco products. In contrast, the European Tobacco Product Directive 2014/40/EU (TPD),^7^ which was transposed into the UK Tobacco and Related Products Regulations 2016 (TRPR),^8^ prohibited characterizing flavors in factory-made and roll-your-own cigarettes across the European Union (EU) and the United Kingdom in May 2016. The transition period for banning the sale of menthol characterizing flavors was delayed until May 2020.

A characterizing flavor is defined in the TPD^7^ and TRPR^8^ as, “a clearly noticeable smell or taste other than one of tobacco, resulting from an additive or a combination of additives, including, but not limited to, fruit, spice, herbs, alcohol, candy, menthol or vanilla, which is noticeable before or during the consumption of the tobacco product.”^8^ Additives that are ’necessary’ for the manufacturing of tobacco products are allowed (eg, sugar when used to replace sugar that is lost during the curing process) provided that the additive does not result in a product with a characterizing flavor.^8^ Neither the TPD^7^ nor TRPR^8^ refers to the maximum menthol content allowed in cigarettes. There is no consensus in sensory science on what is considered “clearly noticeable.” Nevertheless, best practices for identifying characterizing flavors suggest sensory assessments based on comparing the smelling properties of the test product with those of reference products, complemented by chemical analysis of the product^.9,10^

Prior to the UK’s menthol-characterizing flavor ban, in 2018, menthol cigarettes represented 21% of the UK market.^11^ According to survey data, the prevalence of self-reported menthol cigarette smoking among adults in Great Britain declined from 16.2% (October–December 2020) to13.7% (January–March 2023), whereas among 18–24-year-olds, the prevalence declined from 25.7% to 19.4% in this timeframe.^12^ Using a different survey, the prevalence of self-reported menthol smoking among 16-to-19-year in England changed from 12.1% in February 2020 before the characterizing flavor ban was implemented to 3.0% in August 2020 immediately after implementation^13^ and 0.9% in August–September 2021.^14^ A meta-analysis of three real-world studies evaluating the impact of menthol cigarette bans in Canada and The Netherlands reported that 24% of people that smoked menthol cigarettes quit, 50% switched to non-menthol cigarettes, and 24% reported to continue to smoke menthol cigarettes approximately 1 to 2 years after the ban.^15^

In the United Kingdom, the Office for Health Inequalities and Disparities (OHID), is the appointed National Competent Authority that regulates tobacco products. We were commissioned by OHID to conduct sensory testing and chemical analysis on a selection of 20 unburnt cigarette products notified for sale on the UK market to identify if the cigarettes contained a menthol-characterizing flavor. These included 16 ‘test’ products with a potential menthol-characterizing flavor and four ‘reference’ products not expected to have a menthol flavor. The primary aims of this study were: (1) To conduct sensory testing among an untrained consumer panel of adults who smoked daily to identify if they could smell a characterizing menthol/mint flavor, or other non-tobacco flavors; (2) To conduct a chemical analysis to identify and quantify the levels of menthol, other minty flavors, and other flavoring additives in the same cigarette products, and (3) to assess concordance between sensory and chemical assessments.

## Methods

### Study Design

OHID provided a list of products which: (1) tobacco companies actively notified OHID as containing some level of menthol or other minty flavorings (eg, ethyl salicylate, methyl salicylate, eucalyptol, and pulegone), or; (2) were promoted in the trade press as menthol cigarette alternatives, or; (3) had a name suggestive of a menthol-like flavor (eg, “green”), and (4) covered a range of different manufacturers. OHID also provided a list of reference products which did not meet the above criteria. The final 20 products chosen for testing followed an iterative process as some products that OHID had listed, although were still being advertised, were no longer available to purchase.

Cigarette packs were purchased in England from convenience stores or online between October 2021 and April 2022. Each pack was placed unopened, in plastic-lined aluminum foil zip-lock bags and stored in a newly purchased refrigerator, with nothing else in it, until all packs were ready to be prepared and posted to consumer panel members or delivered for chemical analysis.

Sensory and chemical testing established approaches for identifying and quantifying flavor additives in tobacco products,^.9,10,16^ were conducted simultaneously and independently of each other. Sensory testing was conducted by smelling unburnt tobacco using an untrained consumer panel of 50 people who self-reported that they smoked daily. An untrained consumer panel rather than an expert panel was chosen at the request of the funder. The European Commission suggests that a consumer panel can provide information about how “regular consumers” perceive the flavor or odor of tobacco products, though is only useful once data from a sensory panel is combined with analytical data.^9^

Gas chromatography-mass spectrometry (GC-MS) was the selected methodology for chemical assessment. Codes are assigned to cigarette brand names in this paper, as these are part of regulatory procedures currently being undertaken by OHID. The criteria used for determining product non-compliance with the ban on menthol as a characterizing flavor was based on: (1) the proportion of sensory assessments identifying menthol/mint odor was significantly higher compared to reference products and (2) menthol was detected in chemical analysis above the LLOQ.^9,10^

### Sensory Assessments

#### Recruitment and Eligibility Criteria

The consumer panel of 50 participants was recruited via advertising the study through King’s College London, United Kingdom, regional tobacco control networks, and existing smokers’ panels (set up by researchers to enable their research to have involvement from people who smoke).^17,18^ The main purpose of the study (ie, to see if people could detect a characterizing menthol flavor) was not disclosed to panel members in advertising materials, ethical participant information sheets, or smell tests. Participants were therefore blinded and instead advised that the purpose of the study was to determine if people who smoked could smell anything other than tobacco in unburnt cigarettes. Participants were eligible for inclusion if they were aged 18–55 (an upper age cap was included as sensory acuity is known to decline with age); smoked for the past year or longer; smoked ≥5 cigarettes per day; had a computer with camera, microphone, and internet access; not intending to quit smoking in the next 6 months, not pregnant or having plans to get pregnant or breastfeeding and able to provide informed consent (all these inclusion criteria were self-reported). Eligible panel members also had to pass the University of Pennsylvania Brief Smell Identification Test (B-SIT),^19^ which is a booklet containing a 12-item multiple-choice of unique odorants that are released through a pencil scratch of a scent strip. This was posted to potential participants and the test was conducted in a meeting with a member of the research team, carried out via Microsoft Teams with the camera switched on. Potential participants were asked what “the odor smells most like” They had to correctly identify at least 9 of the 12 odorants to be deemed to have a normative olfactory function for someone under 55 years of age^20^ and to be eligible for inclusion in the study. The breakdown of BSIT scores for the 50 participants are included in Table S1.

#### Measures

Sensory assessment of products was conducted with the untrained consumer panel using the Check-All-That-Apply (CATA) test, a validated method recommended for determining characterizing flavors in tobacco products.^9^ CATA fits regulatory needs for characterizing flavors in tobacco products and has been shown to obtain replicable results without training a sensory panel.^21^ Panelists ascertain the presence or absence (yes/no) of specific odor attributes of a product. The intensity of the odor can be indirectly derived from the proportion of consumers that indicate that a particular flavor/odor is present in a tobacco product.

Unlike an expert sensory panel, untrained panels cannot reliably generate a list of odor attributes collectively or discriminate between very specific odors,^9^ so attributes were provided to our consumer panel participants. The 22 attributes used for the CATA test in this study were adapted from a list of 51 attributes generated by an expert EU sensory panel (Table 1 and Table S2).^9^ The EU expert panel list contains names of the odor and corresponding descriptors. Our research team narrowed the list of attributes based on an iterative process that considered which odors may be most known by the study population. For instance, the term “menthol/mint” was used to encompass multiple attributes from the EU list (eg, menthol, menthone, and spearmint).^9^ This approach followed recommendations for using an expert panel to generate relevant vocabulary/attributes that consumer panelists can rely on.^9,10^ The EU list further identifies the possible origin of each odor attribute as a “tobacco odor” or “non-tobacco odor,” and then categorizes types of non-tobacco odors, such as “fruity” (eg, artificial apple, mango, and raspberry).^9^ This was used to define outcome measures of combined categories of odor attributes in this study, specifically any fruity odor, any confectionary odor, and any non-tobacco odor (Table S2).

**Table 1.** Check-All-That-Apply: Cigarette Smell Test Questionnaire

We will ask you to smell each cigarette in a particular order during the call. For each cigarette we ask you to smell, please tell us all attributes that describe the smell			
Odor	Descriptors	Yes/no	Does not know what this is
Artificial apple	Synthetic apple, red apple		
Artificial cherry	Synthetic cherry, cherryade, scented candle, air freshener		
Banana	Fruity, pear drops		
Black tea	Tobacco, blackcurrant, red wine		
Burnt sugar	Caramel, caramelized strawberry		
Butter	Buttermilk, warm milk, movie popcorn		
Cardboard	Dry paper, dry cardboard		
Cheese	Limburger cheese, human sweat, old hops		
Coconut	Shampoo, suntan lotion, Malibu rum		
Dried leaves	Forest floor, winter leaves		
Grape	Grape juice, wine		
Mango	Over-ripe mango, tinned pineapple		
Menthol/mint	Breath freshener, mentholated sweets, peppermint, spearmint, mint, toothpaste		
Orange-limonene	Fresh orange juice, Fanta^TM^ orange		
Peach	Peach, peach skin, crayon		
Prune	Dried fruit, prune juice		
Raisin	Dried fruit, sultanas		
Raspberry	Raspberry juice, raspberry jam		
Rotted dry wood	Decaying tree branches, hay, sweet tobacco		
Smoky	Smoked cheese, smoked ham, smoked fish		
Strawberry milkshake	Strawberry, apple juice, cider		
Vanilla	Ice cream, custard, barrel-aged wine		
Something else not listed			

Adapted from: European Commission, Directorate-General for Health and Food Safety, Methodology to support the decision whether a tobacco product has a characterizing flavor—Application to cigarettes, roll-your-own tobacco, and heated tobacco products, Publications Office of the European Union, 2023, https://data.europa.eu/doi/10.2875/837616.

#### Procedures

Participants were randomized to smell one of the two blocks (A&B) of 10 unburnt cigarettes (eight reference and two reference products) in duplicate (ie, two sets of 25 participants smelled 10 products twice, producing 50 assessments per product). The presentation order of each cigarette was counterbalanced using a Balanced Latin Square of 20 × 20. Having 25 participants smell half the number of products twice, instead of 50 participants smelling 20 products once, enabled us to strike a balance between wanting to understand the reliability of assessment in an untrained panel, the number of products tested, and minimizing participant burden.

When preparing the cigarette samples to be sent out to participants, researchers wore disposable gloves to place each cigarette in its own glass vial bottle, and then clearly numbered them. Product branding on the cigarette rod was masked by black tape. Each sealed vial was placed in an aluminum foil zip-locked bag, placed in a double-walled cardboard box, and posted First Class to each panel member.

The CATA sensory test was conducted remotely in participants’ own homes as for the B-SIT test. A remote setting was chosen due to the ongoing COVID-19 pandemic at the time of the assessments, and this is considered a viable setting for sensory panels.^10^ Panelists were asked to conduct the smell tests alone, in a well-ventilated area of their home without strong odors, such as cigarette smoke or e-cigarette vapor, candles or air fresheners, and to not wear perfume. The researcher directed each participant to select a numbered vial, in the assigned order, and then the researcher verified the number. Participants were instructed to open and sniff the bottle, and then to take the cigarette out and sniff it along the cigarette rod and filter as many times as needed. While sniffing the cigarette, they were asked if they could smell each of the 22 odor attributes, one by one. Participants’ demographics and smoking characteristics/behaviors were recorded at the end of the interview. Figure S1 in Supplementary File 12 is a graphical overview of the study.

#### Analysis

Data analysis was conducted in Stata/SE 16.1 and R version 4.4.1. The degree of association between participants’ first and second smelling was characterized by Yule’s Q.^22^ For the CATA ratings, frequencies and proportions were calculated for assessments that detected each of the 22 individual odor attributes, including menthol/mint, as well as for the following combined categories: any fruit odors, confectionary odors, and any non-tobacco odors. The proportions of assessments from each test product were compared to assess if they were significantly different than the reference products in the same block (ie, non-overlapping 95% Confidence Intervals [CIs] suggested there was a significant difference). This method is recommended by the Health Effects Tobacco Composition (HETOC) Consortium, commissioned by the European Commission^9^ which states “if the attribute is rated significantly higher in a specific test product than in the reference products, thus higher than a ‘noticeable smell’, it can be concluded that this is a ‘clearly noticeable smell.”^9^ In order to account for any dependency in the data due to participants assessing each product twice, proportion tests using cluster-weighted 95% CIs were conducted using the “htestClust” package in R, clustered on participant ID.^23^ Further checks for any influence of within-participant clustering were conducted for the menthol sensory ratings by fitting two regression models with positive menthol sensory detection as the dependent variable and product number as the independent variable; (1) a single logistic regression model with cluster robust standard errors, clustered on participant ID, using the “sandwich” R package,^24^ and (2) a mixed effect model, with participant ID included as a random intercept, using the “lme4” R package.^25^ A further mixed effect model incorporating random slopes failed to converge and so was abandoned.

### Chemical Assessment

#### Preparation

Chemical testing of the same sample of 20 cigarettes was conducted at the Nicotine and Tobacco Product Assessment Resource (NicoTAR) of Roswell Park Comprehensive Cancer Center in Buffalo, New York, USA. Cigarettes were conditioned for at least 48 hours at 22 ± 4°C and 60 ± 2% humidity before sample preparation.^26^ Tobacco was separated from each cigarette with a razor blade, and tobacco filler and non-tobacco material were placed separately in a pre-weighed Erlenmeyer flask. Forty milliliters of a methanol extraction solution containing four internal standards (acenaphthene-d10, chlorobenzene-d5, naphthalene-d8, pyridine-d5, 0.025 mg/ml each) was added to each flask and mixed for 2 hours. Extracts were further diluted by a factor of 10 to mitigate matrix interference. All samples were analyzed in triplicate.

#### Analysis

The content of 34 flavoring agents, including 16 cooling agents were analyzed using a GC-MS method previously reported.^27,28^ The chemicals included in this method were based on commonly reported ingredients in tobacco products such as menthol and synthetic coolants (WS-3 & WS-23).^27–29^ The targeted analyte list, their flavor descriptors, and lower limits of quantitation (LLOQ) are reported in Table S3. Qualitative analysis was performed for other chemicals including non-cooling flavorings by tentatively identifying unknown chromatographic peaks in each sample using laboratory-derived and commercial spectral libraries^30^ following previously published procedures.^27,28^

## Results

### Sensory Assessments

#### Consumer Panel (Participants) Characteristics

Half of the panel participants were males, and 50% were aged 18–24 years. The majority were of White–White British ethnicity (86%), around half (52%) were educated beyond secondary school level and 75% lived in London or Southeast England (Table S4). The majority (76%) smoked between 5 and 10 cigarettes a day (the average number for the whole panel was 9) and had smoked for an average of 12 years (range 1–36; Table S5). Almost half (*n* = 24) also vaped either daily or non-daily, 10 of whom preferred menthol or ice flavors (Table S6).

### Menthol/Mint

#### Sensory Assessment

The majority of sensory assessments for each product were consistent from first to second sensory assessment within each participant (mean proportion of consistent sensory assessments = 81.2%; SD = 5.1%, Yule’s Q = 0.69). Table 2 presents the proportion of sensory assessments identifying a menthol/mint odor. For five test products, the proportion of CATA sensory assessments identifying menthol/mint (range: 38.0%, 95% CI: 23.0% to 53.0% to 58.0%, 95%CI: 42.6% to 73.4%) was significantly higher than the reference products (10%, 95% CI: .4% to 19.6% in block A and 10.0%, 95% CI: 2.2% to 17.8% in block B). The results from the cluster robust and mixed effects regression models did not deviate substantially from those of the proportion tests, although products 10 and 14 were also found to have a significantly higher frequency of assessments identifying menthol/mint than their reference product, but with CIs close to the null (product 10 odds ratios 3.16 [95% CI: 1.19 to 8.43] and 3.65 [95% CI: 1.14 to 11.66]; product 14 odds ratios 3.16 [95% CI: 1.04 to 9.65] and 3.65 [95% CI: 1.14 to 11.66]). See Table S7 and Figure S2 in Supplementary File 13 for full regression results.

**Table 2.** Results From Sensory and Chemical Assessment of Cigarettes (Menthol/Mint)

Product ID	Sensory assessment	Chemical assessment LLOQ = 0.2 mg/cig
	*% (95%CI) of CATA assessments (n = 50) that identified menthol/mint*	*Average amount of menthol* *detected (mg/cig, sd)*
Test products		
1	24.0 (10.3, 37.7)	<LLOQ
2	4.0 (0.0, 9.3)	<LLOQ
**3**	48.0 (33.9, 62.1)*	**0.84 ± 0.04**
**4**	50.0 (34.3, 65.7)*	**1.28 ± (0.05)**
5	16.0 (5.3, 26.7)	<LLOQ
**6**	58.0 (42.6, 73.4)*	**0.95 ± 0.02**
7	38.0 (23.0, 53.0)*	<LLOQ
**8**	48.0 (31.9, 64.1)*	**1.30 ± 0.05**
9	4.0 (0.0, 9.3)	<LLOQ
10	26.0 (11.2, 40.8)	1.03 ± 0.05
11	16.0 (4.0, 28)	<LLOQ
12	12.0 (2.0, 22.0)	<LLOQ
13	14.0 (3.6, 24.4)	<LLOQ
14	26.0 (12.3, 39.7)	1.36 ± 0.05
15	16.0 (6.9, 25.1)	<LLOQ
16	22.0 (10.8, 33.2)	0.46 ± 0.02
Reference products		
17	6.0 (0.0, 12.4)	<LLOQ
18	10.0 (0.4, 19.6)	<LLOQ
19	10.0 (2.2, 17.8)	<LLOQ
20	4.0 (0.0, 9.3)	<LLOQ

CATA = Check-All-That-Apply; 95%CI = 95% Confidence Intervals; * indicates where CATA assessments were significantly higher than the reference product with the highest proportion of assessments (product 18 for products 1–8, and product 19 for products 9–16). See Table S8 for *p*-values from cluster-weighted proportion tests. LLOQ = Lower Limit of Quantitation; SD = Standard Deviation. Emboldened rows show products that had higher CATA assessments than the reference product and contained menthol above the LLOQ.

#### Chemical Assessment

Seven test cigarettes contained menthol levels above the LLOQ (0.2 mg/cig), (ranging from 0.46 to 1.38 mg/cig). Menthol was not detected above the LLOQ for the other test products or for any of the reference products (Table 2). Synthetic coolants were not detected above the LLOQ.

#### Concordance Across the Tests

Four of the sixteen test products had significantly higher proportions of assessments identifying menthol/mint than the reference products in both proportion tests (Table 2) and regression models (Table S7 and Figure S2 in Supplementary File 13) *and* had detectable levels of menthol/mint in the chemical analysis (Table 2; test products 3, 4, 6, 8, emboldened in Table 2).

One product had a significantly higher proportion of assessments identifying menthol/mint than the reference products in both the proportion test and regression models but was below the LLOQ in the chemical assessment (test product 7).

One product had detectable levels of menthol/mint in the chemical analysis, but the proportions of sensory assessments were not significantly higher than the reference product in either the proportion tests or the regression models (test product 16).

Two products had detectable levels of menthol in the chemical analysis and had higher proportions of assessments identifying menthol/mint than reference products in the regression models, but were not significantly higher in the proportion tests (test products 10 and 14).

For the remaining eight test products, there was no significantly higher proportion of sensory assessments detecting menthol/mint compared to the reference products (range 4% to 24%), nor were they significantly higher in the regression models, and these products were below the LLOQ in the chemical analysis (test products 1,2, 5, 9, 11, 12, 13, 15).

### Flavors Other Than Menthol/mint

#### Sensory Testing

Table S9 presents the proportion of smell assessments identifying fruity, confectionary, or non-tobacco (ie, fruity, confectionary, and mint/menthol) odors. A significantly higher proportion of any fruity odors were identified in six test products, a confectionary odor in one test product, and non-tobacco odors in seven test products, compared to the smell assessments for the reference products.

#### Chemical Testing of Other Flavoring Additives

In addition to detecting menthol in seven test products, dihydroxyacetone (which has a simultaneous cooling and sweet taste) was detected in all products, ranging from 4.53 to 13.08 mg/cig. All cigarette products had detectable levels of triacetin (which has a creamy smell/taste and is also used as a plasticizer in the cigarette filter) ranging from 7.84 to 17.76 mg/cig (Table S10). While both chemicals reportedly have sweet and/or cooling properties, it is not clear whether these contributed to sensory attributes other than tobacco flavor. None of the other flavoring agents were detectable above the LLOQ. In the qualitative analysis, humectants propylene glycol and glycerol were detected in 65% (*n* = 13) and 60% (*n* = 12) test products, respectively; several non-cooling flavorings were tentatively identified among test cigarettes (Table S11).

## Discussion

Among an untrained consumer panel of 50 people who currently smoked daily, for five test products, the proportion of CATA smell assessments identifying menthol/mint was significantly higher than the reference products. The chemical analysis identified seven products that contained menthol above the LLOQ; four of these seven products met the criteria for menthol/mint in both sensory and chemical tests. These four products, all from the same manufacturer, therefore appeared to be non-compliant with the UK government’s ban on menthol as a characterizing flavor introduced in 2020. Consumer panels also identified other characterizing flavors, particularly fruity. Untargeted chemical analysis also revealed the presence of several flavorings associated with fruit flavors in one of these products.

In addition to the four products meeting the criteria for menthol/mint in both sensory and chemical tests and consistent across all regression models, there were discordant results for two products—one where menthol/mint was significantly higher than the reference products for the sensory test but was below the LLOQ for the chemical analysis, and one which had detectable levels of menthol/mint in the chemical analysis but low sensory ratings. For a further two products with detectable levels of menthol/mint in the chemical analysis, the findings were inconsistent between the proportion tests and the regression models, hence why we have not made a suggestion about potential compliance. This may suggest that even small quantities of the chemical may still be perceptible to participants (eg, in the case of product 16). It is unknown why a minority of participants detected a menthol odor when the chemical values were below the LLOQ. Possible explanations include—sensory evaluations are subjective, and individual differences between participants (and in our case the lack of training) may have affected how the attributes were perceived.

A significantly higher proportion of fruity odors were identified in six test products and confectionary odor in one test product compared to reference products. For overall non-tobacco odors, seven of the test products were rated significantly higher than the reference products. Although the primary focus of this work was on menthol/mint it would therefore appear that a number of additional products might not be compliant with the legislation which bans all characterizing flavors in cigarettes. However, for flavors other than menthol, there is less correspondence between the sensory and the chemical testing, although some of the products having non-tobacco flavors in the sensory testing had elevated levels of chemicals detected in the qualitative analysis. Among the six products identified as fruity flavors by the panel, we only confirmed the presence of fruity flavorings in a single product. This might be due to limitations of the analytical method used in the study which was developed to target menthol and minty flavorings and only a limited number of other flavoring chemicals. These preliminary findings should be confirmed by further testing of commercial cigarettes. Dihydroxyacetone was detected in all products. While we understand that dihydroxyacetone, similar to hydroxyacetone may be a byproduct of glycerol, glycerol was only detected in 12 (60%) of the 20 products. In contrast to other studies, we did not detect the use of synthetic coolants in our sample of the 20 UK products included in this study. Such substitutes have been identified in cigarettes sold in the United States to comply with state-level menthol regulations.^27,29^

While the majority of the cigarettes tested appeared compliant with the ban on menthol as a characterizing flavor, our findings are consistent with previous literature suggesting that some consumers are still reporting the use of menthol cigarettes after the implementation of a ban.^12–14^ Our findings indicate that menthol is still present in some cigarettes and sufficient for the flavor to be detected by consumers, as identified in other reports.^31^ Nevertheless, given the subjective nature of assessing “characterizing” odors or tastes, banning menthol, related minty flavorings, and other additives not necessary for the manufacturing of tobacco products outright, may be less ambiguous for both regulators and manufacturers and more straightforward to evaluate and enforce.

### Limitations and Strengths

The study has some limitations. We used an untrained consumer panel comprised of adults who smoked cigarettes daily, rather than a trained expert panel.^9,10,16^ Trained and untrained consumer panels each offer unique advantages. An untrained consumer panel has the advantage of not needing a start-up period for screening and training, nor does it require maintenance sessions. They offer real-world insights into consumer sensory perceptions and are more cost-effective than expert panels. However, they lack the detailed sensory profiling and familiarity with specific attributes found in expert panels.^9^ The within-participant findings were reasonably consistent across different modeling techniques. We also did not ask about the recency of nicotine product use or level of craving which may or may not have affected their ability to identify any characterizing flavors. People who smoke have reduced smell acuity.^32^ It is plausible that participants on our panel were less sensitive to a characterizing odor, which could lead to underestimation of some odors. To mitigate this, they all passed the B-SIT.^18^ We used a CATA method for assessing odor presence but did not ask participants to rate odor intensity. This approach is consistent with best practices for untrained consumer panels.^9,10^ We asked the panel to smell rather than taste each product. The disadvantage of smelling tests is the inability to judge characteristics that determine the concept of a flavor such as taste-, nasal- and oral feelings. Some cigarette products may only release a flavor upon combustion,^9^ for example, a smell-only method would likely miss WS compounds, which have a cooling effect but no odor. However, smelling unburnt tobacco is recommended as the preferred starting point, as it captures most of the products with characterizing flavors.^9^ There are also fewer ethical issues related to smelling unburnt tobacco than inhaling combusted tobacco.

Instrument sensitivity was impacted by the tobacco material and it is possible additional flavoring chemicals not reported by our analytical methods may still be present in products with non-tobacco flavors identified by our participants. For example, ethyl salicylate, methyl salicylate, eucalyptol, and pulegone, often added to cigarettes, were not above our lower limits of quantitation. Additionally, while qualitative flavoring identifications were made using commercial spectral libraries, confirmation with analytical standards was not performed. Notwithstanding these limitations, this is one of the few independent published studies that use both sensory and chemical assessments for a menthol/mint characterizing flavor, conducted after a characterizing flavor ban.

## Conclusion

Bans on characterizing flavors in tobacco products, including menthol, were enacted to reduce their appeal. In sensory and chemical tests, we identified four cigarette products for sale in England in 2021–2022 that appeared to be non-compliant with the ban on menthol as a characterizing flavor. However, there was some discordance between the chemical and sensory assessments for four other test products. Banning menthol, other minty flavorings, and additives not essential to the manufacturing process outright, may be less ambiguous for both regulators and manufacturers and more straightforward to evaluate and enforce. In the absence of restrictions on the use of specific additives, characterizing flavor bans may remain subject to exploitation by some manufacturers.

## Supplementary material

Supplementary material is available at *Nicotine and Tobacco Research* online.

ntaf064_suppl_Supplementary_Materials

## Data Availability

The data underlying this article cannot be shared publicly as it contains commercially sensitive information. Parts of the data can be shared on reasonable request to the corresponding author.
