# Supplementary material for: Menthol Characterizing Flavors in Cigarettes on Sale in England After a Characterizing Flavor Ban: Findings From Sensory and Chemical Assessments
Source: Nicotine Tob Res. 2025 Apr 13;27(10):1805–12. doi: 10.1093/ntr/ntaf064 (PMC12453683; doi:10.1093/ntr/ntaf064)

**Supplementary tables**

**Supplementary Table 1. Brief Smell Identification Test (B-SIT) Scores of Panellists used to screen for smell ability.**

| **B-SIT Score Breakdown** | **Block A**  **(n = 25)** | | **Block B**  **(n= 25)** | | **Overall**  **(n = 50)** | |
| --- | --- | --- | --- | --- | --- | --- |
|  | n | % | n | % | n | % |
| **9** | 5 | 20 | 5 | 20 | 10 | 20 |
| **10** | 7 | 28 | 9 | 36 | 16 | 32 |
| **11** | 11 | 44 | 8 | 32 | 19 | 38 |
| **12** | 2 | 8 | 3 | 12 | 5 | 10 |

*A score of 9 or above for a normal sense of smell for 55-year-olds and under is recommended by B-SIT manufacturers.

**Supplementary Table 2: List of 22 odour attributes assessed in the Check-All-That-Apply test by whether or not odour originates from tobacco**

| **Odour** | **Description** | **Odour other than that of tobacco?** | **Type** | **Odour origin** |
| --- | --- | --- | --- | --- |
| Artificial apple | Synthetic apple, red apple | Yes | Fruity | Not a prominent odour in tobacco |
| Artificial cherry | Synthetic cherry, cherryade, scented candle, air freshener | Yes | Fruity | Not a prominent odour in tobacco |
| Banana | Fruity, pear drops | Yes | Fruity | Not a prominent odour in tobacco |
| Black tea | Tobacco, blackcurrant, red wine | No | Tobacco | Chemical breakdown of carotenoid pigments in tobacco leaves during curing |
| Burnt sugar | Caramel, caramelised strawberry | Yes | Confectionary | Not a prominent odour in tobacco |
| Butter | Buttermilk, warm milk, movie popcorn | Yes | Confectionary | Not a prominent odour in tobacco |
| Cardboard | Dry paper, dry cardboard | No | Tobacco | Oxidation of lipids in tobacco leaves during curing |
| Cheese | Limburger cheese, human sweat, old hops | No | Tobacco | Hydrolysis of lipids in tobacco leaves during curing |
| Coconut | Shampoo, suntan lotion, Malibu rum | Yes | Fruity | Not a prominent odour in tobacco |
| Dried leaves | Forrest floor, winter leaves | No | Tobacco | Uncertain origin, but found at noticeable levels in commercial tobacco products |
| Grape | Grape juice, wine | Yes | Fruity | Not a prominent odour in tobacco |
| Mango | Over-ripe mango, tinned pineapple | Yes | Fruity | Not a prominent odour in tobacco |
| Menthol/mint | Breath freshener, mentholated sweets, peppermint, spearmint, mint, toothpaste | Yes | Minty | Not a prominent odour in tobacco |
| Orange-limonene | Fresh orange juice, FantaTM orange | Yes | Fruity | Not a prominent odour in tobacco |
| Peach | Peach, peach skin, crayon | Yes | Fruity | Not a prominent odour in tobacco |
| Prune | Dried fruit, prune juice | No | Tobacco | Uncertain origin, but found at noticeable levels in commercial tobacco products |
| Raisin | Dried fruit, sultanas | No | Tobacco | Uncertain origin, but found at noticeable levels in commercial tobacco products |
| Raspberry | Raspberry juice, raspberry jam | Yes | Fruity | Not a prominent odour in tobacco |
| Rotted dry wood | Decaying tree branches, hay, sweet tobacco | No | Tobacco | Chemical breakdown of carotenoid pigments in tobacco leaves during curing |
| Smoky | Smoked cheese, smoked ham, smoked fish | No | Tobacco | Smoke generated in materials other than tobacco which may be used to dry "fire-cured" tobacco |
| Strawberry milkshake | Strawberry, apple juice, cider | Yes | Fruity | Not a prominent odour in tobacco |
| Vanilla | Ice cream, custard, barrel-aged wine | Yes | Confectionary | Not a prominent odour in tobacco |

Adapted from: European Commission, Directorate-General for Health and Food Safety, Methodology to support the decision whether a tobacco product has a characterising flavour – Application to cigarettes, roll-your-own tobacco and heated tobacco products, Publications Office of the European Union, 2023, <https://data.europa.eu/doi/10.2875/837616>

**Supplementary Table 3 – Flavouring chemicals and limits of quantitation targeted in quantitative analysis**

| Chemical | CAS Number | Flavour Type | LLOQ (mg/cig) | Chemical | CAS Number | Flavour Type | LLOQ (mg/cig) |
| --- | --- | --- | --- | --- | --- | --- | --- |
| 1,4-Cineole | 470-67-7 | Cooling | *0.10* | Isopulegol | 7786-67-6 | Minty | *0.20* |
| 2,3,5-Trimethylpyrazine | 14667-55-1 | Musty | *0.10* | Isovanillin | 621-59-0 | N/A | *4.00* |
| 5-(hydroxymethyl)furfural | 67-47-0 | Herbal | *2.00* | Limonene | 138-86-3 | Terpenic | *0.10* |
| Acetoin | 513-86-0 | Creamy | *2.00* | Linalool | 78-70-6 | Citrus | *0.20* |
| Benzaldehyde | 100-52-7 | Fruity | *0.10* | Maltol | 118-71-8 | Caramellic | *4.00* |
| Benzyl Alcohol | 100-51-6 | Fruity | *0.20* | Menthol | 1490-04-6 | Cooling | *0.20* |
| Butanoic Acid | 107-92-6 | Sour | *2.00* | Menthone | 14073-97-3 | Minty | *0.10* |
| Carvone | 99-49-0 | Minty | *0.10* | Menthyl Acetate | 16409-45-3 | Minty | *0.10* |
| Cinnamaldehyde | 14371-10-9 | Spicy | *0.40* | Methyl Salicylate | 119-36-8 | Minty | *0.20* |
| Dihydroxyacetone | 96-26-4 | Cooling | *2.00* | Nicotine | 54-11-5 | N/A | *0.20* |
| Ethyl Maltol | 4940-11-8 | Caramellic | *2.00* | Piperitone | 89-81-6 | Minty | *0.20* |
| Ethyl Salicylate | 118-61-6 | Minty | *0.20* | Pulegone | 89-82-7 | Minty | *0.10* |
| Ethyl Vanillin | 121-32-4 | Vanilla | *2.00* | Raspberry Ketone | 5471-51-2 | Berry | *0.20* |
| Eucalyptol | 470-82-6 | Minty | *0.10* | Triacetin | 102-76-1 | Creamy | *0.20* |
| Eugenol | 97-53-0 | Spicy | *0.20* | Vanillin | 121-33-5 | Vanilla | *2.00* |
| Fenchol | 1632-73-1 | Camphoreous | *0.10* | WS-3 | 39711-79-0 | Cooling | *0.20* |
| Furaneol | 3658-77-3 | Caramellic | *2.00* | WS-23 | 51115-67-4 | Minty | *0.20* |
| Isomenthol | 20752-33-4 | Cooling | *0.20* | --- | --- | --- | --- |

Flavour descriptors provided by the Good Scents Company.

LLOQ=Limit of Quantitation

LLOQ values were determined from method validation. The lowest concentration level in the calibration that was within ±20% of the actual recovery over five batches was selected as the LLOQ. , For some chemicals, performance at the lower end was less precise and therefore LLOQs were elevated.

**Supplementary Table 4. Demographic characteristic of the consumer panel**

| Demographics | Overall (N = 50)  % (n) | Block A  (n = 25) | Block B  (n = 25) |
| --- | --- | --- | --- |
| **Gender** |  |  |  |
| Male | 50% (25) | 56% (14) | 44% (11) |
| Female | 48% (24) | 44% (11) | 52% (13) |
| Non-binary | 2% (1) | 0% (0) | 4% (1) |
| **Age** | | | |
| 18-24 | 50% (25) | 52% (13) | 48% (12) |
| 25-29 | 22% (11) | 28% (7) | 16% (4) |
| 30-39 | 14% (7) | 12% (3) | 16% (4) |
| 40-49 | 6% (3) | 4% (1) | 8% (2) |
| 50-55 | 8% (4) | 4% (1) | 12% (3) |
| **Ethnicity** |  |  |  |
| Asian/Asian British | 6% (3) | 12% (3) | 0% (0) |
| Black/Black British | 0% (0) | 0% (0) | 0% (0) |
| White/White British | 86% (43) | 76% (19) | 96% (24) |
| Mixed/Multiple ethnic groups | 6% (3) | 8% (2) | 4% (1) |
| Other ethnic groups | 2% (1) | 4% (1) | 0% (0) |
| **Region** |  |  |  |
| East of England | 2% (1) | 0% (0) | 4% (1) |
| London | 28% (14) | 24% (6) | 32% (8) |
| North East England | 14% (7) | 0% (0) | 28% (7) |
| South East England | 46% (23) | 72% (18) | 20% (5) |
| South West England | 2% (1) | 0% (0) | 4% (1) |
| Scotland | 4% (2) | 0% (0) | 8% (2) |
| West Midlands | 2% (1) | 4% (1) | 0% (0) |
| Yorkshire | 2% (1) | 0% (0) | 4% (1) |
| **Educational attainment** |  |  |  |
| Secondary school advanced | 8% (4) | 8% (2) | 8% (2) |
| Further education below degree level | 34% (17) | 52% (13) | 16% (4) |
| Currently at university | 16% (8) | 0% (0) | 32% (8) |
| Completed undergraduate degree | 16% (8) | 12% (3) | 20% (5) |
| Post-graduate degree (MSc/PhD) | 26% (13) | 28% (7) | 24% (6) |

**Supplementary Table 5: Smoking characteristics of the consumer panel**

| Characteristic | Overall (N = 50) | Block A (N = 25) | Block B (N = 25) |
| --- | --- | --- | --- |
| **Usual tobacco product** |  |  |  |
| Factory-made cigarettes | 30% (15) | 24% (6) | 36% (9) |
| Roll-your-own cigarettes | 30% (15) | 28% (7) | 32% (8) |
| Both | 40% (20) | 48% (12) | 32% (8) |
| **Own preferred product included in their sensory assessment** |  |  |  |
| No | 90% (45) | 84% (21) | 96% (24) |
| Yes | 10% (5) | 16% (4) | 4% (1) |
| **Number of cigarettes smoked per day** | |  |  |
| 5 | 32% (16) | 24% (6) | 40% (10) |
| 6-9 | 34% (17) | 36% (9) | 32% (8) |
| 10 | 10% (5) | 12% (3) | 8% (2) |
| 11-14 | 6% (3) | 8% (2) | 4% (1) |
| 15-19 | 10% (5) | 8% (2) | 12% (3) |
| 20 | 8% (4) | 12% (3) | 4% (1) |
| **Time to first cigarette of the day** |  |  |  |
| Within 5-minutes | 8% (4) | 12% (3) | 4% (1) |
| 6 to 30 minutes | 22% (11) | 20% (5) | 24% (6) |
| 31 to 60 minutes | 22% (11) | 20% (5) | 24% (6) |
| 60 minutes | 48% (24) | 48% (12) | 48% (12) |
| **Ever tried to quit** |  |  |  |
| No | 22% (11) | 16% (4) | 28% (7) |
| Yes | 78% (39) | 84% (21) | 72% (18) |
| **Number of years spent smoking** | |  |  |
| 1-4 | 12% (6) | 4% (1) | 20% (5) |
| 5-9 | 42% (21) | 56% (14) | 28% (7) |
| 10 | 12% (6) | 12% (3) | 12% (3) |
| 11-14 | 8% (4) | 4% (1) | 12% (3) |
| 15-20 | 12% (6) | 16% (4) | 8% (2) |
| 20-29 | 6% (3) | 4% (1) | 8% (2) |
| 30-40 | 8% (4) | 4% (1) | 12% (3) |
| **Current use of other nicotine products*** |  |  |  |
| None | 40% (20) | 40% (10) | 40% (10) |
| E-cigarettes/vapes | 48% (24) | 48% (12) | 48% (12) |
| Cigars/cigarillos | 4% (2) | 0% (0) | 8% (2) |
| Heated tobacco | 2% (1) | 4% (1) | 0% |
| Snus | 2% (1) | 4% (1) | 0% |

* Some panel member used more than one product

**Supplementary Table 6: Vaping characteristics of the consumer panel**

| Characteristic | Overall  (N = 50) | Block A  (N = 25) | Block B  (N = 25) |
| --- | --- | --- | --- |
| **Frequency of e-cigarette use** |  |  |  |
| Current daily | 28% (14) | 36% (9) | 20% (5) |
| Current non-daily | 20% (10) | 12% (3) | 28% (7) |
| Has tried a vape once/few times | 30% (15) | 32% (8) | 28% (7) |
| Ex-vaper | 6% (3) | 8% (2) | 4% (1) |
| Never vaped | 16% (8) | 12% (3) | 20% (5) |
| **Preferred e-liquid flavours (n=24)*** |  |  |  |
| Fruits | 66.6% (18) | 37% (10) | 29.6% (8) |
| Ice flavours (cooling without mint) | 7.4% (2) | 4.7% (1) | 4.7% (1) |
| Menthol/Mint | 33.3% (9) | 18.5% (5) | 14.8% (4) |
| Sweet Desserts & Confectionary  (ice cream, cheesecake & bubble gum) | 11.1% (3) | 7.4% (2) | 4.7% (1) |
| Soft Beverages  (cola, energy drinks & pink lemonade) | 22.2% (6) | 7.4% (2) | 14.8% (4) |
| Tobacco | 14.8% (4) | 11.1% (3) | 4.7% (1) |

*Panel members could report use of more than one flavour

**Supplementary Table 7: Regression results (Outcome=Menthol identified in sensory tests)**

|  | Model 1: cluster-robust SEs | | Model 2: Random intercept | |
| --- | --- | --- | --- | --- |
| Product | OR (95% CI) | p-value | OR (95% CI) | p-value |
| *Ref = product 18* | - | - | - | - |
| product 1 | 2.84 (0.73 - 11.09) | 0.133 | 3.17 (0.98 - 10.29) | 0.055 |
| product 2 | 0.38 (0.10 - 1.38) | 0.140 | 0.36(0.06 - 1.99) | 0.240 |
| **product 3** | **8.31 (2.19 - 31.49)** | **0.002** | **11.1 (3.55 - 34.7)** | **<0.001** |
| **product 4** | **9.00 (2.41 - 33.63)** | **0.001** | **12.21(3.90 - 38.21)** | **<0.001** |
| product 5 | 1.71 (0.51 - 5.77) | 0.384 | 1.80 (0.52 - 6.20) | 0.353 |
| **product 6** | **12.43 (3.01 - 51.28)** | **<0.001** | **17.94 (5.67 - 56.75)** | **<0.001** |
| **product 7** | **5.52 (1.57 - 19.35)** | **0.008** | **6.83 (2.18 - 21.41)** | **0.001** |
| **product 8** | **8.31 (2.35 - 29.35)** | **0.001** | **11.10 (3.55 - 34.7)** | **<0.001** |
| *Ref = product 19* | - | - | - | - |
| product 9 | 0.38 (0.06 - 2.22) | 0.279 | 0.35 (0.07 - 1.93) | 0.230 |
| **product 10** | **3.16 (1.19 - 8.43)** | **0.021** | **3.65 (1.14 - 11.66)** | **0.029** |
| product 11 | 1.71 (0.41 - 7.18) | 0.461 | 1.81 (0.53 - 6.18) | 0.343 |
| product 12 | 1.23 (0.32 - 4.69) | 0.765 | 1.25 (0.35 - 4.52) | 0.734 |
| product 13 | 1.47 (0.36 - 5.99) | 0.595 | 1.52 (0.43 - 5.32) | 0.512 |
| **product 14** | **3.16 (1.04 - 9.65)** | **0.043** | **3.65 (1.14 - 11.66)** | **0.029** |
| product 15 | 1.71 (0.58 - 5.05) | 0.328 | 1.81 (0.53 - 6.18) | 0.343 |
| product 16 | 2.54 (0.90 - 7.14) | 0.077 | 2.83 (0.87 - 9.20) | 0.084 |

OR = Odds ratio, CI = Confidence interval. Model 1 is a logistic regression with cluster-robust standard errors (participant ID as the clustering variable). Model 2 is a generalised linear mixed effect model with a logit link, and a random intercept for each participant ID.

**Supplementary Table 8. Proportion tests, including p-values, clustered on participant id. (Menthol sensory assessments)**

| Product number | Percent | LCI | UCI | p-value |
| --- | --- | --- | --- | --- |
| 1 | 24.0 | 10.3 | 37.7 | 0.529 |
| 2 | 4.0 | 0.0 | 9.3 | <0.001* |
| **3** | **48.0** | **33.9** | **62.1** | **<0.001** |
| **4** | **50.0** | **34.3** | **65.7** | **<0.001** |
| 5 | 16.0 | 5.3 | 26.7 | 0.509 |
| **6** | **58.0** | **42.6** | **73.4** | **<0.001** |
| **7** | **38.0** | **23.0** | **53.0** | **0.016** |
| **8** | **48.0** | **31.9** | **64.1** | **<0.001** |
| 9 | 4.0 | 0.0 | 9.3 | <0.001* |
| 10 | 26.0 | 11.2 | 40.8 | 0.277 |
| 11 | 16.0 | 4.0 | 28.0 | 0.770 |
| 12 | 12.0 | 2.0 | 22.0 | 0.258 |
| 13 | 14.0 | 3.6 | 24.4 | 0.474 |
| 14 | 26.0 | 12.3 | 39.7 | 0.241 |
| 15 | 16.0 | 6.9 | 25.1 | 0.700 |
| 16 | 22.0 | 10.8 | 33.2 | 0.462 |
| 17 | 6.0 | 0.0 | 12.4 | <0.001 |
| 18 | 10.0 | 0.4 | 19.6 | 0.050 |
| 19 | 10.0 | 2.2 | 17.8 | 0.050 |
| 20 | 4.0 | 0.0 | 9.3 | <0.001 |

* p-values are two sided so products 2 and 9 have significant p-values (alongside products 3,4,6,7 and 8), as detection of menthol lower than reference products. Reference products: product 18 for products 1-8, and product 19 for products 9-16

**Supplementary Table 9: results from sensory assessment (fruity, confectionary and non- tobacco odours)**

| Test Products | *% (95%CI) of CATA assessments (n=50) that identified any fruit^1^odours* | *% (95%CI) of CATA assessments (n=50) that identified any confectionary odours^2^* | *% (95%CI) of CATA assessments (n=50) that identified any non-tobacco odour^3^* |
| --- | --- | --- | --- |
| 1 | 36.0 (20.8, 51.2) | 30.0 (15.3, 44.7) | 66.0 (48.7, 83.3) |
| 2 | 26.0 (12.3, 39.7) | 40.0 (25.3, 54.7) | 50.0 (34.3, 65.7) |
| 3 | 56.0 (40.0, 72.0)* | 26.0 (12.3, 39.7) | 84.0 (72.0, 96.0)* |
| 4 | 54.0 (39.4, 68.6)* | 30.0 (16.4, 43.6) | 82.0 (69.8, 94.2)* |
| 5 | 20.0 (7.6, 32.4) | 34.0 (20.7, 47.3) | 56.0 (43.2, 68.8) |
| 6 | 70.0 (56.4, 83.6)* | 22.0 (9.5, 34.5) | 92.0 (84.8, 99.2)* |
| 7 | 34.0 (24.9, 43.1) | 40.0 (25.3, 54.7) | 78.0 (65.5, 90.5) |
| 8 | 48.0 (33.9, 62.1) | 26.0 (12.3, 39.7) | 76.0 (64.7, 87.3) |
| 9 | 26.0 (13.5, 38.5) | 56.0 (43.2, 68.8)* | 70.0 (60.4, 79.6)* |
| 10 | 68.0 (55.8, 80.2)* | 26.0 (13.5, 38.5) | 80.0 (68.9, 91.1)* |
| 11 | 32.0 (19.8, 44.2) | 26.0 (13.5, 38.5) | 62.0 (49.3, 74.7) |
| 12 | 28.0 (16.8, 39.2) | 24.0 (10.3, 37.7) | 54.0 (40.5, 67.5) |
| 13 | 30.0 (17.6, 42.4) | 36.0 (23.0, 49.0) | 64.0 (51.0, 77.0) |
| 14 | 56.0 (41.0, 71.0)* | 24.0 (10.3, 37.7) | 76.0 (63.5, 88.5)* |
| 15 | 44.0 (27.1, 60.9) | 12.0 (3.6, 20.4) | 60.0 (44.3, 75.7) |
| 16 | 62.0 (48.1, 75.9)* | 18.0 (8.6, 27.4) | 72.0 (58.3, 85.7)* |
| **Reference Products** |  |  |  |
| 17 | 24.0 (10.3, 37.7) | 22.0 (9.5, 34.5) | 40.0 (25.3, 54.7) |
| 18 | 20.0 (7.6, 32.4) | 30.0 (15.3, 44.7) | 50.0 (33.4, 66.6) |
| 19 | 24.0 (14.2, 33.8) | 12.0 (3.6, 20.4) | 40.0 (27.6, 52.4) |
| 20 | 20.0 (6.4, 33.6) | 14.0 (2.2, 25.8) | 36.0 (20.8, 51.2) |

1 artificial apple, artificial cherry, banana, coconut, grape, mango, orange-limonene, peach, raspberry, strawberry milkshake). 2 (burnt sugar, butter, vanilla). 3 fruit, confectionary and menthol/mint combined. * Significantly different than the reference product in the respective block with the highest proportion of assessments that identified the respective odour (i.e., non-overlapping 95% CIs suggested there was a significant difference).

**Supplementary Table 10: Determined content of Dihydroxacetone and Triacetin**

| Product code | Dihydoxyacetone: Average amount detected: mg/cig (SD) | Triacetin: Average amount detected: mg/cig (SD) |
| --- | --- | --- |
| Test Products |  |  |
| 1 | 5.54 (0.57) | 10.71 (0.60) |
| 2 | 5.72 (0.50) | 9.67 (0.62) |
| 3 | 5.75 (0.54) | 10.28 (2.78) |
| 4 | 6.87 (1.00) | 11.33 (0.98) |
| 5 | 8.27 (0.79) | 13.21 (1.23) |
| 6 | 6.83 (0.60) | 16.15 (0.67) |
| 7 | 5.40 (0.36) | 13.00 (0.79) |
| 8 | 5.82 (0.72) | 10.45 (0.66) |
| 9 | 6.37 (0.32) | 7.84 (1.95) |
| 10 | 4.79 (0.65) | 10.62 (1.01) |
| 11 | 7.60 (1.07) | 8.91 (3.72) |
| 12 | 8.78 (0.19) | 10.52 (0.82) |
| 13 | 6.18 (0.60) | 9.40 (0.90) |
| 14 | 6.13 (0.40) | 10.24 (1.35) |
| 15 | 7.67 (1.26) | 17.76 (1.19) |
| 16 | 4.53 (0.40) | 10.11 (0.79) |
| Reference Products |  |  |
| 17 | 6.19 (0.69) | 11.95 (0.22) |
| 18 | 5.59 (0.09) | 9.99 (0.52) |
| 19 | 7.64 (0.58) | 11.05 (0.18) |
| 20 | 13.08 (0.43) | 9.26 (0.43) |

**Supplementary Table 11: qualitative identification of other flavouring additives**

|  | Propylene glycol  (57-55-6) | Glycerol  (56-81-5) | Allyl alcohol  (107-18-6) | Hydroxyacetone  (116-09-6) | Butyl formate  (592-84-7) | α-Angelica lactone  (591-12-8) | Citronellyl butyrate  (141-16-2) | 1,3-Dioxolane, 2-(1-ethylpentyl)-  (4359-47-1) |
| --- | --- | --- | --- | --- | --- | --- | --- | --- |
|  | Sweet | Sweet | Pungent, mustard | Sweet, slightly green, burnt | Fruity, plum, rum, brandy | Sweet, creamy, coconut | Fruity, sweet, waxy | Fruity, radish, chrysanthemum |
| **Test Products** | --- | --- | --- | --- | --- | --- | --- | --- |
| 1 | --- | --- | --- | --- | --- | --- | --- | --- |
| 2 | --- | --- | --- | --- | --- | --- | --- | --- |
| 3 | **X** | **X** | --- | --- | --- | --- | --- | --- |
| 4 | **X** | **X** | --- | --- | --- | --- | --- | --- |
| 5 | **X** | --- | --- | **X** | --- | --- | --- | --- |
| 6 | **X** | **X** | **X** | --- | --- | --- | --- | --- |
| 7 | **X** | --- | --- | --- | --- | --- | --- | --- |
| 8 | **X** | **X** | --- | --- | --- | --- | --- | --- |
| 9 | **X** | --- | --- | **X** | --- | --- | --- | --- |
| 10 | **X** | **X** | --- | --- | --- | --- | --- | --- |
| 11 | --- | **X** | --- | --- | --- | --- | --- | --- |
| 12 | --- | --- | **X** | --- | --- | --- | --- | --- |
| 13 | --- | --- | --- | --- | --- | --- | --- | **X** |
| 14 | **X** | **X** | --- | --- | --- | --- | --- | --- |
| 15 | **X** | **X** | --- | --- | --- | --- | --- | --- |
| 16 | **X** | **X** | --- | --- | **X** | --- | **X** | --- |
| **Reference Products** | --- | --- | --- | --- | --- | --- | --- | --- |
| 17 | --- | --- | --- | --- | --- | --- | --- | --- |
| 18 | **X** | **X** | --- | --- | --- | --- | --- | --- |
| 19 | --- | **X** | --- | --- | --- | --- | --- | --- |
| 20 | **X** | **X** | **X** | --- | --- | **X** | --- | --- |
|  |  |  |  |  |  |  |  |  |
|  |  |  |  |  |  |  |  |  |
|  |  |  |  |  |  |  |  |  |
|  |  |  |  |  |  |  |  |  |
|  |  |  |  |  |  |  |  |  |

**X** indicates tentative identification based on 1) a laboratory-derived, 2) National Institute for Standards and Technology (NIST 2017), 3) Flavors and Fragrances of Natural and Synthetic Compounds (FFNSC, 3rd edition) spectral libraries. We applied a match factor of 70% to tentative identifications from the NIST. Samples were analysed in triplicate, and tentative identifications over 70% are retained if found in at least two replicates.

Flavour descriptors (odour and/or taste) provided by the Good Scents Company.

**Figure 1, Supplementary file 12: Graphic overview of the study**

GCMS testing performed on all products…


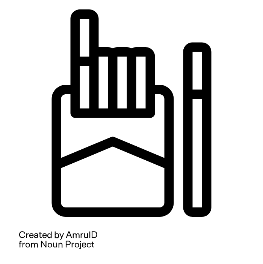

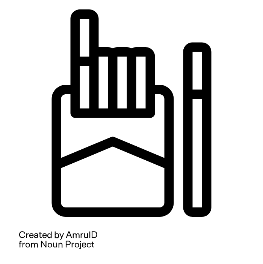


Each group was allocated 10 products for sensory testing (8 test and 2 reference), which was conducted twice per group with the presentation order changed between tests

Participants (N=50) were divided into two groups

8 test products

2 reference products

8 test products

2 reference products

**Participants**

**Products**

**GCMS testing**


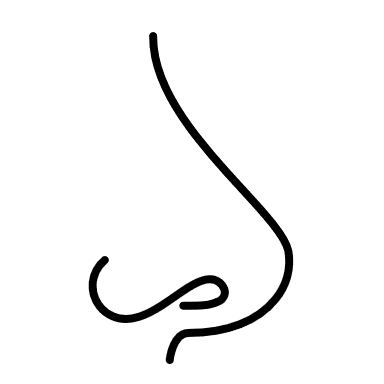


n=25


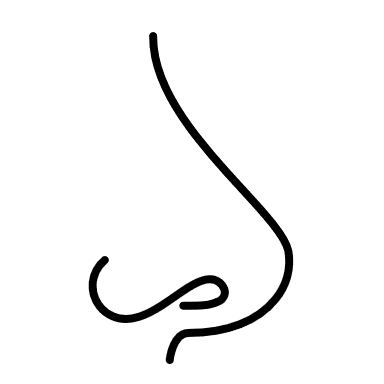


n=25


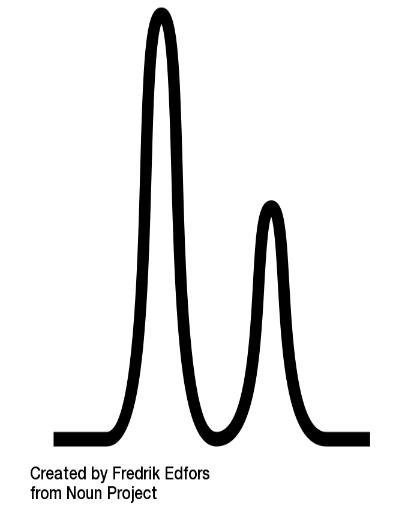


**Figure 2: Supplementary file 13: Lattice plot comparing ORs and 95%CIs from two regression models**


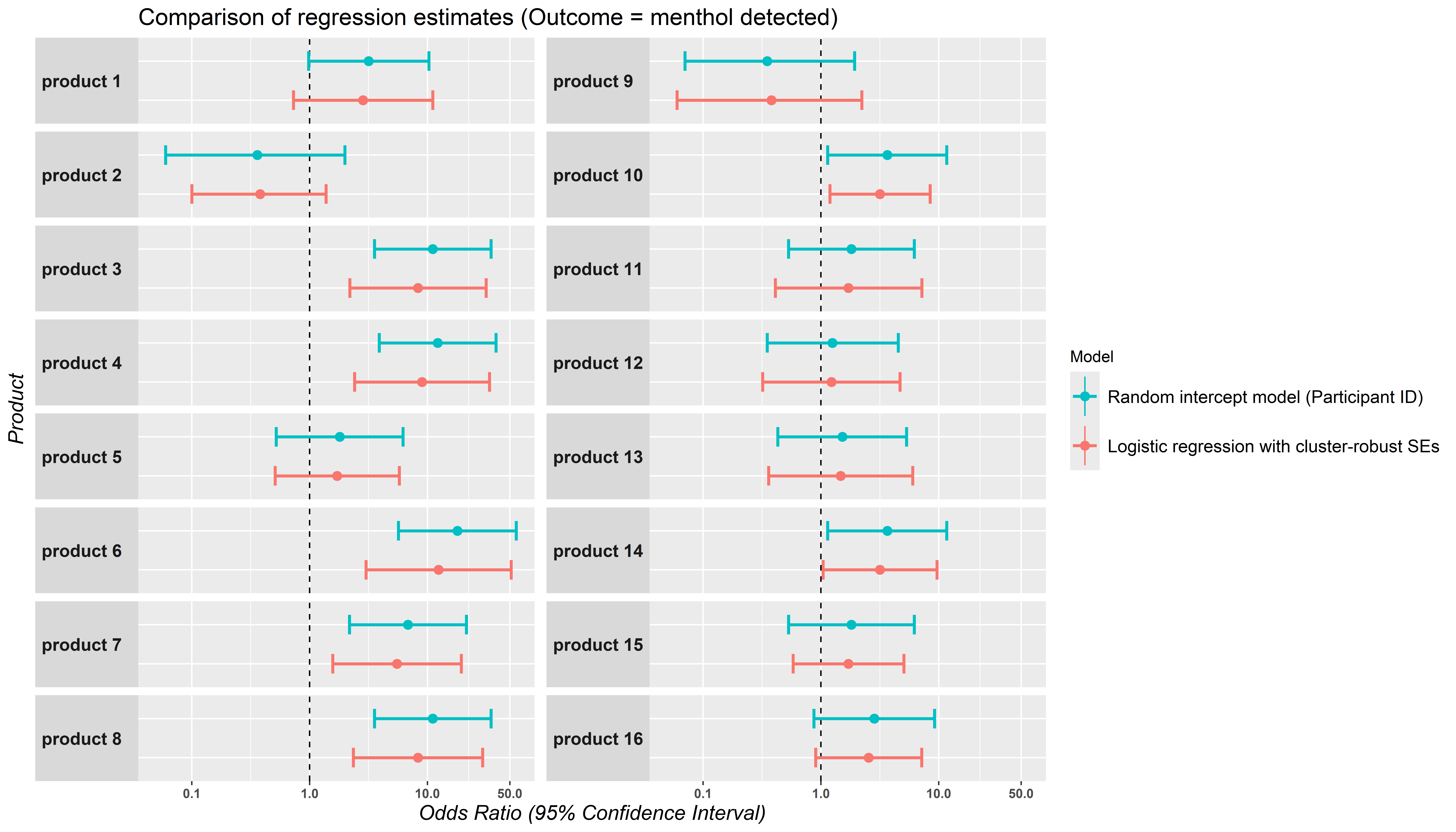

Supplement: ntaf064_suppl_Supplementary_Materials [file ntaf064_suppl_supplementary_materials.docx]
